# Inhibition of eNOS Partially Blunts the Beneficial Effects of Nebivolol on Angiotensin II-Induced Signaling in H9c2 Cardiomyoblasts

**DOI:** 10.3390/cimb44050144

**Published:** 2022-05-10

**Authors:** Rukhsana Gul, Nouf Alsalman, Assim A. Alfadda

**Affiliations:** 1 Obesity Research Center, College of Medicine, King Saud University, P.O. Box 2925, Riyadh 11461, Saudi Arabia; nouf.ibrahim.92@gmail.com (N.A.); aalfadda@ksu.edu.sa (A.A.A.); 2 Department of Medicine, College of Medicine, King Saud University, P.O. Box 2925, Riyadh 11461, Saudi Arabia; 3 Strategic Center for Diabetes Research, College of Medicine, King Saud University, P.O. Box 2925, Riyadh 11461, Saudi Arabia

**Keywords:** reactive oxygen species, nitric oxide, nebivolol, angiotensin II, H9c2 cardiomyoblasts

## Abstract

We have recently illustrated that nebivolol can inhibit angiotensin II (Ang II)-mediated signaling in cardiomyoblasts; however, to date, the detailed mechanism for the beneficial effects of nebivolol has not been studied. Here, we investigated whether the inhibition of NO bioavailability by blocking eNOS (endothelial nitric oxide synthase) using L-NG-nitroarginine methyl ester (L-NAME) would attenuate nebivolol-mediated favorable effects on Ang II-evoked signaling in H9c2 cardiomyoblasts. Our data reveal that the nebivolol-mediated antagonistic effects on Ang II-induced oxidative stress were retreated by concurrent pretreatment with L-NAME and nebivolol. Similarly, the expressions of pro-inflammatory markers TNF-α and iNOS stimulated by Ang II were not decreased with the combination of nebivolol plus L-NAME. In contrast, the nebivolol-induced reduction in the Ang II-triggered mTORC1 pathway and the mRNA levels of hypertrophic markers ANP, BNP, and β-MHC were not reversed with the addition of L-NAME to nebivolol. In compliance with these data, the inhibition of eNOS by L-N⁵-(1-Iminoethyl) ornithine (LNIO) and its upstream regulator AMP-activated kinase (AMPK) with compound C in the presence of nebivolol showed effects similar to those of the L-NAME plus nebivolol combination on Ang II-mediated signaling. Pretreatment with either compound C plus nebivolol or LNIO plus nebivolol showed similar effects to those of the L-NAME plus nebivolol combination on Ang II-mediated signaling. In conclusion, our data indicate that the rise in NO bioavailability caused by nebivolol via the stimulation of AMPK/eNOS signaling is key for its anti-inflammatory and antioxidant properties but not for its antihypertrophic response upon Ang II stimulation.

## 1. Introduction

The stimulation of the renin–angiotensin–aldosterone system (RAAS) enhances the activation of angiotensin II (Ang II), a peptide linked to cardiovascular diseases (CVDs) [1,2,3,4,5,6]. The binding of Ang II to AT1R elicits inflammatory cytokines and growth-promoting signaling cascades, which sequentially causes cardiovascular maladaptation [1,2,3,4]. Earlier studies have demonstrated that the Ang II-stimulated ROS increase caused by NADPH oxidase plays a crucial role in disrupting redox networks, triggering adverse cardiovascular problems [6,7,8]. Nitric oxide (NO) is an effective vasorelaxant required to maintain cardiovascular and metabolic homeostasis [9,10,11]. It is produced from L-arginine, molecular oxygen, and reduced NADPH via an enzymatic reaction driven by nitric oxide synthase (NOS). Under normal physiological conditions, the generation of NO regulates blood pressure, and it may impact cardiac function and remodeling. The activation of eNOS and the sequential increase in NO bioavailability are downregulated by an increase in superoxide generation. The drop in NO bioavailability is a prime indicator of ROS provoked by Ang II [10]. Endothelial dysfunction provoked by a NO and Ang II imbalance gives rise to tissue injury by oxidizing biological macromolecules, such as lipids and proteins, thereby instigating the progression of CVDs [10,11].

The activation of the β3-adrenergic receptor induces negative inotropic effects opposite to those of the β1-adrenergic receptor and the β2-adrenergic receptor by stimulating the NOS pathway [12]. The agonistic activation of the β3-adrenergic receptor improves acute myocardial ischemia–reperfusion injury, while its deficiency leads to exacerbated remodeling to pressure-induced heart failure. These beneficial properties of β3-adrenergic receptor stimulation have been mainly ascribed to the stimulation of AMPK/eNOS signaling and the consequent increase in NO bioavailability [13,14,15]. The inhibition of the β1-adrenergic receptor is known to enhance contractile function by increasing NO bioavailability [16,17]. Indeed, nebivolol is a selective third-generation β1-adrenergic antagonist, which, unlike conventional β-blockers, was found to shut down NADPH oxidase activation and ROS in a rodent model treated with Ang II [18]. Nebivolol also stimulates the β3-adrenergic receptor to synthesize NO via AMPK/eNOS activation, which promotes vasodilatation and enhances endothelial function [17]. The inhibition of the β3-adrenergic receptor with a pharmacological inhibitor has been found to significantly abrogate nebivolol-evoked cardiac NO production, signifying a potent function of the β3-adrenergic receptor in NO generation by nebivolol [19,20]. Thus, it is conceivable that nebivolol offers dual cardiac protection by both blocking the β1-adrenergic receptor and stimulating the β3-adrenergic receptor/AMPK/eNOS pathway.

Ang II-induced amplification in ROS and diminution in NO bioavailability activate redox-sensitive genes, which augment inflammatory and growth factors [21]. We have previously revealed that nebivolol represses the Ang II-triggered mammalian target of the rapamycin complex1 (mTORC1) activation and the microRNA expression of miR-208a in HL-1 cardiomyocytes [3]. We have also very recently delineated the positive effects of nebivolol on Ang II signaling in H9c2 cells [22]. These pleotropic effects of nebivolol on Ang II-induced signaling might be due to the suppression of oxidative stress via β3-adrenergic receptor stimulation, which subsequently activates AMPK/eNOS to create a surge in bioavailable NO. In view of the aforementioned evidence, we hypothesize that the repression of Ang II-provoked signaling caused by nebivolol in cardiomyocytes could be due to its tendency to increase the bioavailable NO via eNOS activation [18,19]. To assess our hypothesis, nebivolol effects on Ang II-mediated signaling in H9c2 cardiomyoblasts were tested in the presence of an eNOS antagonist, L-NAME. We also measured the mRNA levels of Ang II-stimulated growth and pro-inflammatory factors in H9c2 cells treated with nebivolol plus L-NAME. Furthermore, we assessed the influence of the eNOS inhibitor L-N⁵-(1-Iminoethyl) ornithine (LNIO) and the AMPK inhibitor compound C plus nebivolol on Ang II-stimulated signaling in H9c2 cells.

## 2. Materials and Methods

### 2.1. Materials

Ang II and nebivolol were obtained from Sigma-Aldrich (St. Louis, MO, USA), and L-NAME was obtained from Calbiochem (La Jolla, CA, USA). Antibodies for phospho-mTOR (pSer2448), mTOR, phospho-p70S6K (pThr389), p70S6K, phospho-RPS6 (pSer235/236), and RPS6 were obtained from Cell Signaling Technology Inc. (Boston, MA, USA). ECL substrates for high-sensitivity Western blot detection, Pageruler plus prestained ladder, and SuperScript™ III First-Strand Synthesis System were obtained from Thermo Fisher Scientific (Waltham, MA, USA). TRIzol™ Reagent was obtained from Invitrogen (Waltham, MA, USA), and PowerUp SYBR Green master mix was obtained from Applied Biosystems (Thermo Fisher Scientific, Waltham, MA, USA).

### 2.2. Cell Culture and Western Blotting

H9c2 cardiomyoblasts derived from rat embryonic cardiomyocytes were obtained from the American Type Culture Collection (ATCC) (Manassas, VA, USA, cat no: CRL-1446TM). Cultures were grown on Dulbecco’s Modified Eagle Medium (DMEM, ATCC 30-2002), supplemented with fetal bovine serum (FBS) (10%) (ATCC, 30-2020) and penicillin/streptomycin (100 U/mL: 100 mg/mL). Cells were cultured in an incubator with a 37 °C temperature and 5% CO2-air. The cultures were passaged after 70–80% confluence was achieved. For Western blotting, cells were collected using trypsinization after treatment. Lysis buffer was added, and samples were lysed on ice as demonstrated previously [1,2,3,4]. For immunoblotting, membranes were probed with mTOR, phospho-mTOR (pSer2448), p70S6K, phospho-p70S6K (pThr389), RPS6, and phospho-RPS6 (pSer235/236) antibodies.

Binding of antibody to membrane was detected using ECL substrates, and images were captured using G: BOX (Syngene, Cambridge, UK). Protein band density was quantified using Gene Tools image analysis software (Syngene, Cambridge, UK). The data are presented as the normalized protein band density in arbitrary units.

### 2.3. Quantitation of NADP^+^/NADPH

The intracellular levels of NADP+ and NADPH were determined by employing colorimetric assay using NADP^+^/NADPH Assay Kit (ab65349, Abcam, Cambridge, MA, USA) as per manufacturer specifications. After assay, as per kit instructions, the absorbances of NADP+ and NADPH were measured at OD450 nm. The concentrations of NADP+ and NADPH were calculated as per the given protocol.

### 2.4. RNA Extraction and Real-Time Polymerase Chain Reaction

TRIzol reagent was used for the extraction of RNA from H9c2 cells as per the manufacturer guide. RNA quantity was determined using Nano drop (Thermo Scientific™ Nano Drop 2000c). Reverse transcription was used to synthesize cDNA using 400 ng of RNA per 20 µL reaction using SuperScript™ III First-Strand Synthesis System. Real-time-PCR was carried out by using RT-PCR detection System (CFX96 Touch™ Real-Time PCR Detection System-Bio-Rad) using SYBR Green PCR master mix (Applied Biosystems, Waltham, MA, USA). Target mRNA levels were normalized to control levels, and relative levels of mRNA were calculated by using the 2^−ΔΔCt^ method [22]. The expression of β-actin (housekeeping gene) was also evaluated and used as an internal control. The primers (Table 1) were obtained from Macrogen Inc., (Seoul, Korea).

### 2.5. Measurement of Intracellular ROS

The redox-sensitive dye 2,7-dichlorodihydrofluorescein diacetate (DCFDA; Molecular Probe, Eugene, OR, USA) was employed to measure generation of ROS in cells [6]. H9c2 cardiomyoblasts were pretreated with the redox-sensitive dye DCFDA (4 μM) for a period of 20 min. After incubation, cells were washed with DPBS and pre-incubated with inhibitors for 40 min at 37 °C, prior to Ang II treatment. DCFDA fluorescence was measured using a plate reader, Synergy HT multi-mode reader (BioTek Instruments, Inc., Winooski, VT, USA), at excitation and emission wavelengths of 488 and 520 nm, respectively. For fluorescence measurements, after incubation, cells were washed and incubated with complete culture medium. Live-cell images were acquired using a fluorescent cell imager (Floid Cell Imaging Solution; Thermo Fisher Scientific, Waltham, MA, USA). The data were attained from at least three to five independent experiments, and the fluorescence intensity is expressed as fold change vs. untreated control.

### 2.6. Measurement of Cell Surface Area

After treatment with Ang II and inhibitors for 48 h, cells were washed with PBS and fixed with 4% paraformaldehyde for 10 min. Cells were then stained with 1% Crystal violet (Sigma) for 10 min. Six to eight images were acquired randomly from each group using Zeiss AxioCam MRc5 color camera attached to ZEISS Axio Observer inverse microscope (Carl Zeiss MicroImaging GmbH, Wetzlar, Germany). The cell surface area of nearly 100 cells was measured using Zen 2 lite software (Carl Zeiss Microscopy, Wetzlar, Germany).

### 2.7. Statistical Analysis

Data represent means ± SEM of at least three to six separate experiments. Differences in the multiple groups were compared using analysis of variance (ANOVA) followed by Student’s *t*-test. Two-group analysis was performed using Student’s *t*-test (paired or unpaired as appropriate). A value of *p* < 0.05 is considered significant.

## 3. Results

### 3.1. Nebivolol-Promoted NO Bioavailability Is Critical to Reduce Ang II-Triggered Oxidative Stress in H9c2 Cardiomyoblasts

**Figure 1:**
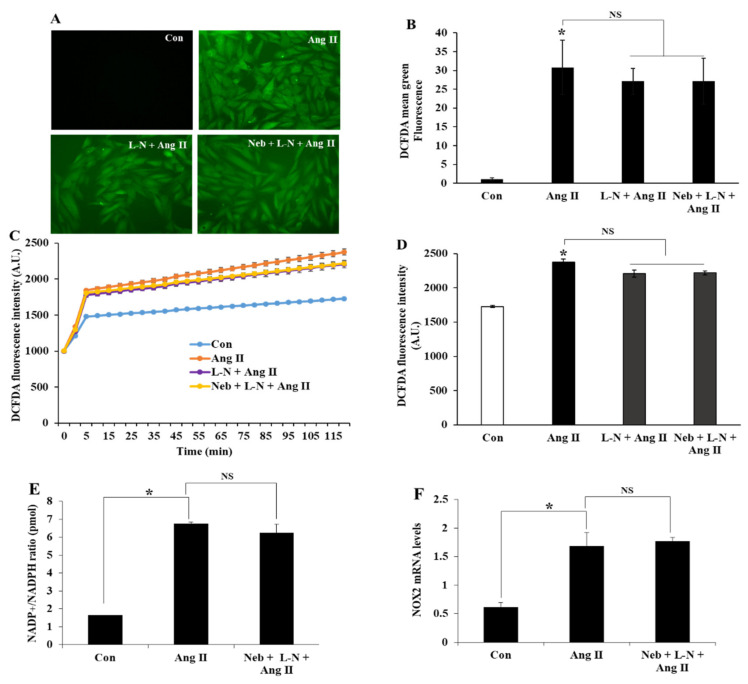
Representative DCF fluorescence images and analysis of ROS production in H9c2 cells pretreated with L-NAME (L-N) (100 µM) or L-N plus nebivolol (Neb) (1 µM) prior to Ang II stimulation (**A**,**B**). Ang II (1 µM) increased the DCFDA fluorescence, which was not affected by either L-N or L-N plus Neb (**C**,**D**). The quantification of fluorescence changes in DCFDA-loaded cells using a fluorescence reader at an excitation and emission of 488 and 522 nm, respectively. Ang II-stimulated an increase in NADP^+^/NADPH ratio, and NOX2 mRNA expression levels were not reduced by pretreatment with L-N plus Neb (**E**,**F**). * *p* < 0.05 vs. untreated (Con) and NS, nonsignificant vs. Ang II. Values are means ± SEM N ≥ 6 for each treatment group.

Increases in NO bioavailability via the nebivolol-mediated activation of eNOS reduce oxidative stress [20]. To evaluate the involvement of NO in nebivolol-mediated beneficial effects, we measured Ang II-triggered ROS and NADPH oxidase activity in the presence or absence of the eNOS inhibitor L-NAME (100 µM) plus nebivolol (1 µM). H9C2 cells treated with Ang II (1 µM) exhibited a significant increase in DCFDA fluorescence compared to the control (Figure 1A–D). Pretreatment with L-NAME or L-NAME plus nebivolol did not alter Ang II-triggered DCFDA fluorescence. These data indicate that NO bioavailability caused by nebivolol is a prerequisite to ameliorate Ang II-triggered oxidative damage in H9c2 cardiomyoblasts.

Conceptually, Ang II could possibly augment oxidative stress or increase ROS production by enhancing the NADP^+^/NADPH ratio. Previous studies have demonstrated NADPH oxidase as a dominant site for superoxide synthesis in response to Ang II stimulation in H9c2 cells [22]. To further demarcate the function of NO bioavailability in nebivolol-mediated protective effects, we tested the effects of the L-NAME plus nebivolol combination on NADP^+^ and NADPH, and then we measured the NADP^+^/NADPH ratio. Compared to the untreated control, Ang II elevated the NADP^+^/NADPH ratio in H9c2 cardiomyoblasts. Consistent with the above observation, pretreatment with the L-NAME plus nebivolol combination had no influence on Ang II-stimulated NADPH oxidase activity (Figure 1E). Taken together, these data indicate that the diminution of NO bioavailability caused by the eNOS inhibitor L-NAME could be accountable for the ineffectiveness of nebivolol to mitigate the NADP^+^/NADPH ratio triggered by Ang II. We further evaluated the role of the L-NAME plus nebivolol combination on NOX2, the main subunit of NADPH oxidase, by evaluating its mRNA expressions. Consistent with the above observations, a rise in NOX2 mRNA expression was observed upon Ang II stimulation, which was not suppressed by pretreatment with the nebivolol plus L-NAME combination (Figure 1F). These data demonstrate that NO is important for the nebivolol-mediated beneficial effects in Ang II-induced superoxide production and NADPH oxidase activation in H9c2 cardiomyoblasts.

### 3.2. Nebivolol-Mediated NO Production Is Not Critical to Reduce Ang II-Triggered Hypertrophy Response

**Figure 2:**
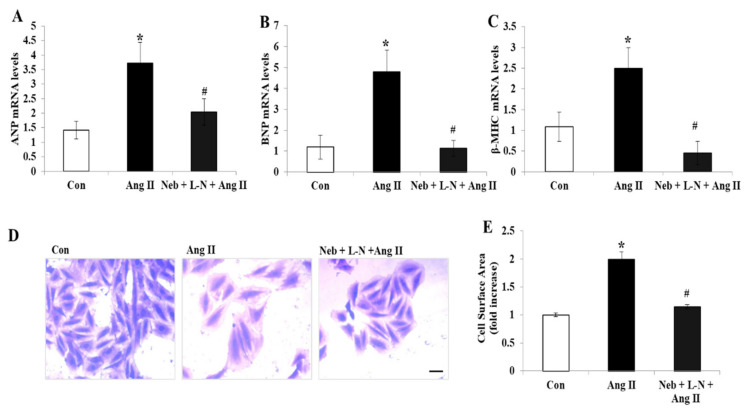
Effects of Neb plus L-N combination on Ang II-induced mRNA expression of cardiac hypertrophy genes in H9c2 cardiomyoblasts. Downregulation of mRNA expression of atrial natriuretic peptide (ANP) (**A**), brain natriuretic peptide (BNP) (**B**), and β-myosin heavy chain (β-MHC) (**C**) by Neb + L-N. (**D**) Images representing morphological changes in H9c2 cells stained with crystal violet; scale bar = 20 μm. (**E**) Cell surface area was analyzed and is presented as mean (n = 100 cells/group). * *p* < 0.05 vs. untreated (Con) and # *p* < 0.05 vs. Ang II. Values are means ± SEM. N ≥ 3–6 for each treatment group.

To further assess the role of NO bioavailability caused by nebivolol in Ang II signaling, we evaluated cellular hypertrophy. We have previously revealed the antagonistic effects of nebivolol on Ang II-mediated hypertrophy marker expression in H9c2 cardiomyoblasts. The exposure of H9c2 cells to Ang II for 48 h remarkably augmented cardiac hypertrophy genes, such as atrial natriuretic peptide (ANP), B-type natriuretic peptide (BNP), and β-myosin heavy chain (β-MHC) (Figure 2A–C). Additionally, an increase in the H9c2 cell surface area was observed after Ang II exposure for 48 h (Figure 2D,E).

Interestingly, in contrast to our above observations, pretreatment with the L-NAME plus nebivolol combination noticeably diminished the gene expression levels of ANP, BNP, and β-MHC and significantly reduced the cell surface area in H9c2 cardiomyoblasts (Figure 2A–E). These findings indicate that the rise in NO bioavailability caused by nebivolol is not critical for the prevention of the hypertrophic response induced by Ang II in H9c2 cardiomyoblasts.

### 3.3. Nebivolol-Mediated NO Bioavailability Is Not Required for Attenuation of Ang II-Induced Kinase Activation

**Figure 3:**
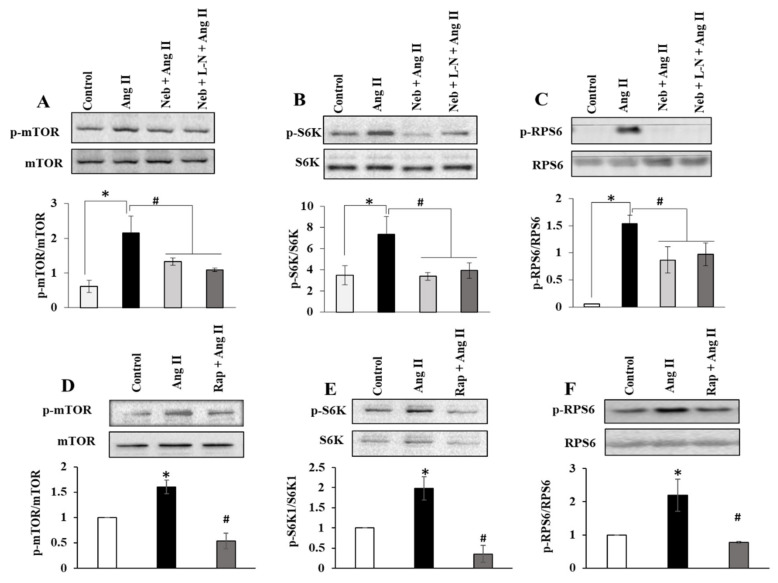
Effects of Neb plus L-N combination on Ang II-induced mTOR signaling in H9c2 cardiomyoblasts. Top: Representative images of autoradiograms showing elevated levels of pSer2448 mTOR (**A**), pThr389 S6K1 (**B**), and pSer235/236RPS6 (**C**) in H9c2 cardiomyoblasts stimulated with Ang II and suppression by Neb and Neb + L-N. Bottom: Graphs show results of densitometry analysis of the intensity of the phosphorylated protein bands after adjusting for the intensity of total protein bands (tmTOR, tS6K1, tRPS6). Top: Representative images of autoradiograms showing elevated levels of pSer2448 mTOR (**D**), pThr389 S6K1 (**E**), and pSer235/236RPS6 (**F**) in H9c2 cardiomyoblasts stimulated with Ang II and suppression by Rap. Bottom: Graphs show results of densitometry analysis of the intensity of the phosphorylated protein bands after adjusting for the intensity of total protein bands (tmTOR, tS6K1, tRPS6). * *p* < 0.05 vs. untreated (Con) and # *p* < 0.05 vs. Ang II. Values are means ± SEM. N ≥ 3–6 for each treatment group.

Ang II-induced cardiac hypertrophic growth is known to increase by the deregulation of mTORC1 signaling. Thus, to further demonstrate the influence of L-NAME and nebivolol co-treatment on Ang II signaling, we compared the impact of nebivolol and the nebivolol plus L-NAME combination on Ang II mediated on the mTORC1 pathway by determining mTOR, S6K1, and RPS6 phosphorylation levels. Similarly, to nebivolol, the nebivolol plus L-NAME combination exhibited a substantial reduction in the phosphorylation of mTOR, S6K1, and RPS6 (Figure 3A–C), suggesting that the elimination of NO by L-NAME has no influence on the Ang II-induced activation of the mTORC1 signaling pathway. Additionally, the Ang II-induced phosphorylation of mTOR, S6K1, and RPS6 were reduced by pretreatment with an inhibitor of mTORC1 rapamycin (10 nM) (Figure 3D–F). These data indicate that nebivolol-mediated prophylactic effects on Ang II signaling in H9c2 cardiomyoblasts are partially reliant on its ability to enhance the bioavailability of NO.

### 3.4. Nebivolol-Mediated NO Bioavailability Is Essential for Suppression of Cardiac Proinflammatory Cytokines Induced by Ang II

**Figure 4:**
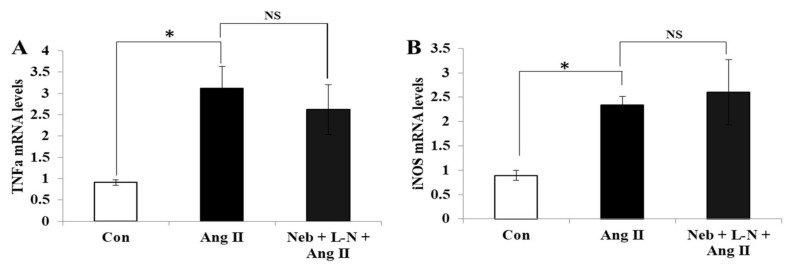
Effects of Neb plus L-N combination on Ang II-induced mRNA expression of proinflammatory cytokines in H9c2 cardiomyoblasts. mRNA levels of TNF-α (**A**) and iNOS (**B**) were not reduced by Neb + L-N. * *p* < 0.05 vs. untreated (Con) and NS, nonsignificant vs. Ang II. Values are means ± SEM. N ≥ 3–6 for each treatment group.

The activation of pro-inflammatory cytokines by Ang II plays a marked role in the advancement of CVD [23]. To further expand on the effects of eNOS inhibition on the nebivolol-mediated positive effects on Ang II-incited pathology, we assessed the anti-inflammatory effects of the combination of nebivolol plus L-NAME on TNF-α and iNOS mRNA expressions using RT-PCR. As shown in Figure 4A,B, the Ang II-provoked increases in TNF-α and iNOS mRNA levels in H9c2 were not reduced by the L-NAME plus nebivolol combination but, in fact, were markedly elevated compared to those of the control. These data highlight the notion that NO bioavailability is a requisite for nebivolol-driven anti-inflammatory effects on Ang II-stimulated H9c2 cells.

### 3.5. Effects of Compound C plus Nebivolol Combination on Ang II-Triggered ROS and mRNA Levels of Inflammatory and Hypertrophy Markers

**Figure 5:**
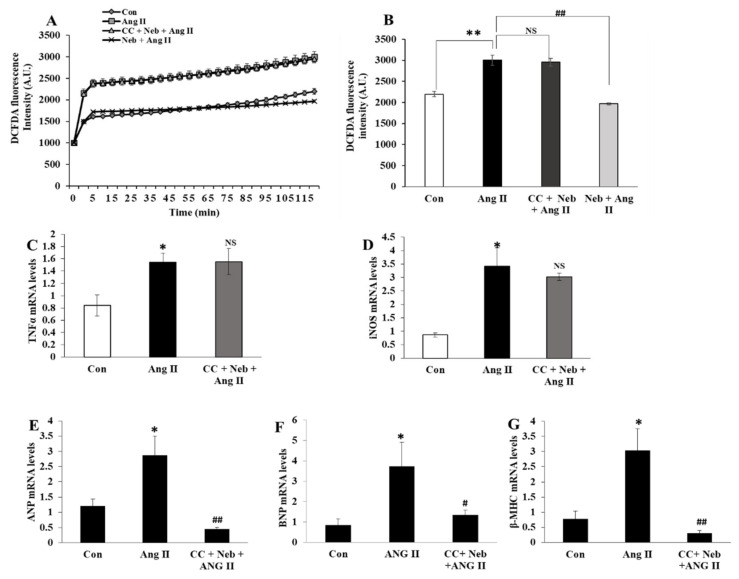
Effects of Neb plus compound C (CC) on Ang II-induced ROS generation and mRNA expressions of hypertrophy and inflammatory genes. Ang II-induced increase in DCFDA fluorescence was not affected by pretreatment with CC (5 µM) plus Neb (1 µM) but was significantly reduced by Neb alone (**A**,**B**) Ang II-mediated mRNA expression levels of proinflammatory cytokines TNF-α (**C**) and iNOS (**D**) were not reduced by CC plus Neb pretreatment. Ang II-mediated mRNA expressions of hypertrophy markers ANP (**E**), BNP (**F**), and β-MHC (**G**) were significantly reduced by CC plus Neb pretreatment. ** *p* < 0.02, * *p* < 0.05 vs. untreated (Con). ## *p* < 0.02, # *p* < 0.05 vs. Ang II and NS, nonsignificant vs. Ang II. Values are means ± SEM N ≥ 6 for each treatment group.

To determine whether the inhibition of AMPK, an upstream kinase regulator of eNOS would produce effects similar to those of eNOS inhibitors on Ang II signaling, we evaluated the effects of the AMPK inhibitor, the compound C plus nebivolol combination, on Ang II-provoked ROS formation and the transcription of inflammatory and hypertrophy genes in H9c2 cardiomyoblasts. Pretreatment of H9c2 cardiomyoblasts with compound C plus nebivolol did not reduce ROS or the gene expressions of TNF-α and iNOS induced by Ang II (Figure 5A–D). However, the mRNA levels of hypertrophy genes ANP, BNP and β-MHC, were downregulated by the compound C plus nebivolol combination (Figure 5E–G). These findings affirm that the rise in NO bioavailability caused by nebivolol is critical to decrease ROS and proinflammatory cytokines but not for the suppression of hypertrophy provoked by Ang II in H9c2 cardiomyoblasts.

### 3.6. Effects of LNIO plus Nebivolol Combination on Ang II-Induced ROS Generation and mRNA Expressions of Inflammatory and Hypertrophic Genes

To further confirm the above observation, we next evaluated the effects of a different inhibitor of eNOS, the LNIO plus nebivolol combination, on Ang II-induced signaling. In accordance with our above findings, pretreatment of H9c2 cardiomyoblasts with LNIO plus nebivolol did not alter Ang II-induced ROS generation in H9c2 cardiomyoblasts (Supplementary Figure S1A,B). Similarly, Ang II-induced mRNA expression levels of TNF-α and iNOS did not change, but the levels of BNP and β-MHC were significantly reduced by the LNIO plus nebivolol combination (Supplementary Figure S1C–F). Taken together, these data further demonstrate that eNOS activation is required for the anti-inflammatory and antioxidant effects of nebivolol but not for its anti-hypertrophy effects on Ang II stimulation.

## 4. Discussion

Here, we demonstrate that the inhibition of Ang II-mediated signaling by nebivolol is partially dependent on its bioavailable NO production in H9c2 cardiomyoblasts. We also demonstrate that NO bioavailability caused by nebivolol is not vital for the inhibition of hypertrophy but that it is compulsory for the suppression of ROS and the proinflammatory actions triggered by Ang II. Nebivolol is a third-generation β1-adrenergic receptor antagonist, and its vasodilator properties have been demonstrated to have a hemodynamic profile, which provides beneficial effects in its approved use in hypertension and heart failure [20,22,24]. The stimulation of eNOS and the scavenging of superoxide by nebivolol occur in a synergetic manner to provide cardiovascular protection. Thus, compared to conventional β-blockers, these beneficial features make nebivolol a better drug of choice to control hypertension, heart failure, and other CVDs [25]. Treatment with nebivolol in different rodent models has been linked to the depletion of ROS and NADPH oxidase activity and, thus, to reduced cardiac fibrosis. The ROS scavenging properties of nebivolol are associated with the inhibition of NADPH oxidase and β3-adrenergic receptor/AMPK/eNOS cascade activation [26]. We have previously shown the novel pleiotropic effects of nebivolol on Ang II-provoked pathological pathways in HL-1 cardiomyocytes and H9c2 cardiomyoblasts [3,22]. Other studies have recently revealed that nebivolol improves aortic remodeling in hypertensive rats, where hypertension was induced by L-NAME, and these positive effects were incited by eNOS upregulation and ROS inhibition [27]. The cardioprotective effects of nebivolol on myocardial injury were notably reduced by L-NAME, indicating that nebivolol prevents cardiac dysfunctions through eNOS-mediated NO synthesis. Mercanoglu et al. also demonstrated that the protective effects of nebivolol in cardiorenal syndrome were mainly due to a reduction in nitrosative damage [28]. Additionally, the activation of NOS signaling is associated with the antihypertrophic effects of nebivolol in neonatal cardiomyocytes [29]. Besides its β1-adrenergic receptor antagonistic action, the binding of nebivolol to the β3-adrenergic receptor plays a key role in increasing vasodilatation and decreasing vascular resistance or by countering the negative consequences arbitrated by overactivation of the β1-adrenergic receptor [19,24]. The stimulation of the β3-adrenergic receptor by nebivolol induces AMPK/eNOS/NO cascade activation.

The growing evidence supports the notion that the over-activation of RAAS triggers a redox imbalance, thereby eliciting ROS and decreasing NO production. NO reduces the expression of AT1R, and the eNOS inhibitor promotes AT1R expression, indicating that NO might act as an inhibitory regulator of AT1R expression [30]. Furthermore, NO modulates protein functions and inhibits the activation of protein kinases triggered by Ang II [7,11]. We have also recently demonstrated the role of nebivolol in Ang II-triggered ROS and NADPH oxidase activation. Accordingly, we posit that the NO-donating properties of nebivolol account for its pleiotropic effects on the inflammatory and growth promoting signaling triggered by Ang II. Therefore, to understand this phenomenon, we used a combination of L-NAME plus nebivolol and assessed Ang II-mediated signaling in H9c2 cardiomyoblasts. In the current study, we observed that Ang II-provoked ROS and the NADP^+^/NADPH ratio were not suppressed by treatment with either L-NAME or nebivolol plus L-NAME. Thus, it is conceivable that nebivolol-mediated inhibitory actions in Ang II-mediated specific effects are regulated by eNOS activation.

Despite such compelling evidence, Ang II-mediated mTOR and the phosphorylation of its downstream signaling molecules RPS6 and S6K1 were decreased almost to the same extent in both pretreatments, nebivolol alone and nebivolol plus L-NAME. The deregulation of mTORC1 signaling is anticipated to induce myocardial hypertrophy [31], which is connected with the re-expression of fetal genes. In the hypertrophic response to Ang II stimulation, cardiac cells typically respond by upregulating ANP, BNP, and β-MHC levels. We observed a significant rise in ANP, BNP, and β-MHC mRNA levels in Ang II-treated cells compared to in the control; however, the levels were markedly reduced in cells treated with either nebivolol or nebivolol plus L-NAME. These data were further confirmed when assessing the morphological changes in cells. The ang II-induced increase in cell size was inhibited by pretreatment with nebivolol plus L-NAME. Surprisingly, these results do not support our proposition pertaining to the key role of NO generation by nebivolol in restoring cardiac function in response to Ang II stimuli.

Our results were further validated by using an inhibitor of AMPK, compound C, and a selective pharmacological inhibitor of eNOS, LNIO, in combination with nebivolol on Ang II-provoked signaling. AMPK is an upstream serine/threonine kinase known to phosphorylate eNOS at Ser-1177 and stimulate NO synthesis [32]. The activation of the β3-adrenergic receptor in cardiomyocytes protects from myocardial remodeling by triggering the AMPK signaling pathway [33]. In agreement with our above findings, the Ang II-induced rise in superoxide formation was not reduced by compound C plus nebivolol. Furthermore, the expressions of genes for hypertrophy, such as ANP, BNP, and β-MHC, were decreased in pretreatment with compound C plus nebivolol. Similar results were obtained for treatment with LNIO plus nebivolol against Ang II-induced ROS generation and the mRNA expressions of hypertrophy genes. These findings indicate the existence of a mechanism that does not require the activation of the AMPK/eNOS pathway by nebivolol. Our currents findings are in accordance with our previous reports showing that AMPK or eNOS inhibition does not completely revert the impact of nebivolol on Ang II signaling [3,34].

The mounting evidence demonstrates that Ang II modulates the expressions of pro-inflammatory mediators in the myocardium, which is attributed to hypertension and CVDs [35,36]. In particular, proinflammatory markers, such as TNF-α and iNOS, are upregulated in cardiovascular disease [35,37,38]. We investigated the relevance of our above observations by measuring Ang II-provoked TNF-α and iNOS gene expressions. We observed a substantial decline in both TNF-α and iNOS mRNA levels caused by nebivolol in response to Ang II. Unexpectedly, in our parallel observation of the effect of the nebivolol plus L-NAME combination on Ang II-provoked redox signaling, neither TNF-α nor iNOS gene expressions were reduced by the nebivolol plus L-NAME combination. Likewise, pretreatment with LNIO plus nebivolol or compound C plus nebivolol had no influence on Ang II-triggered TNF-α and iNOS gene expressions. Thus, together, these data confirm the critical and beneficial role of increased NO biosynthesis caused by nebivolol in Ang II-provoked ROS and chronic inflammatory responses.

## 5. Conclusions

**Figure 6:**
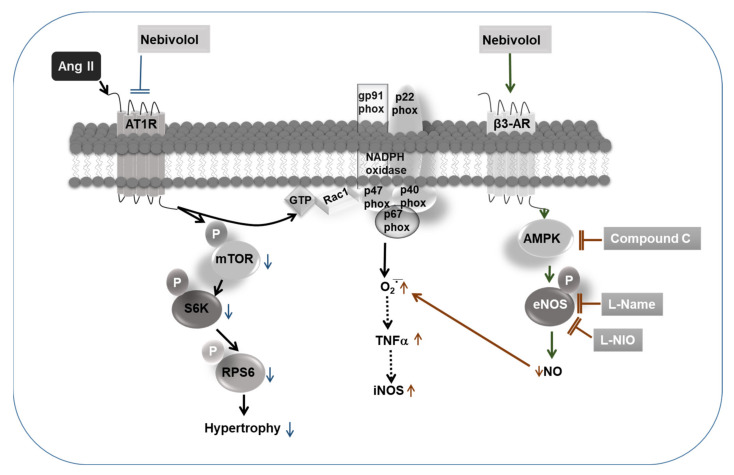
Schematic diagram showing that the protective effects of nebivolol on Ang II-induced signaling are partially blunted by inhibition of eNOS and AMPK. NO production via the agonistic activation of β3-AR/AMPK/eNOS pathway by nebivolol is required for its inhibitory effects on Ang II-induced ROS generation and activation of proinflammatory cytokines, such as TNF-α and iNOS. On the contrary, inhibitory effects of nebivolol on Ang II-mediated mTORC1 signaling and hypertrophic response are completely independent of NO bioavailability.

Our data suggest that the inhibition of NO bioavailability caused by antagonizing eNOS or its activator AMPK alleviates the defensive effects of nebivolol on the Ang II-stimulated increase in ROS formation. Additionally, our data reveal that the nebivolol-promoted increase in NO bioavailability is vital for its anti-inflammatory effects on Ang II stimulation. The findings from this study reveal for the first time that eNOS suppression, by either pharmacological inhibitors eNOS or AMPK, does not downregulate the cardioprotective effects of nebivolol on Ang II-triggered hypertrophy (Figure 6). These findings provide new insights into the prevailing effects of nebivolol and indicate the existence of an eNOS-independent mechanism of the nebivolol-mediated beneficial effects on Ang II-induced cardiac pathology.

## Figures and Tables

**Table 1:** Primer sequences.

Gene	Forward and Reverse Primers 5′-3′
ANP	AAAGCAAACTGAGGGCTCTGCTCG
	TTCGGTACCGGAAGCTGTTGCA
BNP	ACAATCCACGATGCAGAAGC
	CGCCGATCCGGTCTATCTTC
β-MHC	ATCTACAGCGGGTGAAGCAG
	CAGGTTAGCCTTGGCCTTGA
TNF-α	CACTCAGGCATCGACATTCG
	CACCGGCAAGGATTCCAA
NOX2	TGAATCTCAGGCCAATCACTTT
	AAT GGTCTTGAACTCGTTATCCC
iNOS	CGGCCACCAGCTTCTTCA
	TGCTTACAGGTCTACGTTCAAGACAT
β-actin	CAA CGT CAC ACT TCA TGA TGG A
	ATG CCC CGA GGC TCT CTT

## Supplementary Materials

The following supporting information can be downloaded at: https://www.mdpi.com/article/10.3390/cimb44050144/s1.
