# Supplementary material for: Inhibition of eNOS Partially Blunts the Beneficial Effects of Nebivolol on Angiotensin II-Induced Signaling in H9c2 Cardiomyoblasts
Source: Curr Issues Mol Biol. 2022 May 10;44(5):2139–52. doi: 10.3390/cimb44050144 (PMC9164031; doi:10.3390/cimb44050144)
Supplement: Supplementary file 1 [file cimb-44-00144-s001.zip › cimb-1647562-supplementary.pdf]

Supplementary Data

Inhibition of eNOS Partially Blunts the Beneficial Effects of Nebivolol on Angiotensin II-Induced Signaling in H9c2 Cardiomyoblasts

Rukhsana Gul [1], *, Nouf Alsalman [1] and Assim A. Alfadda [1,2,3]

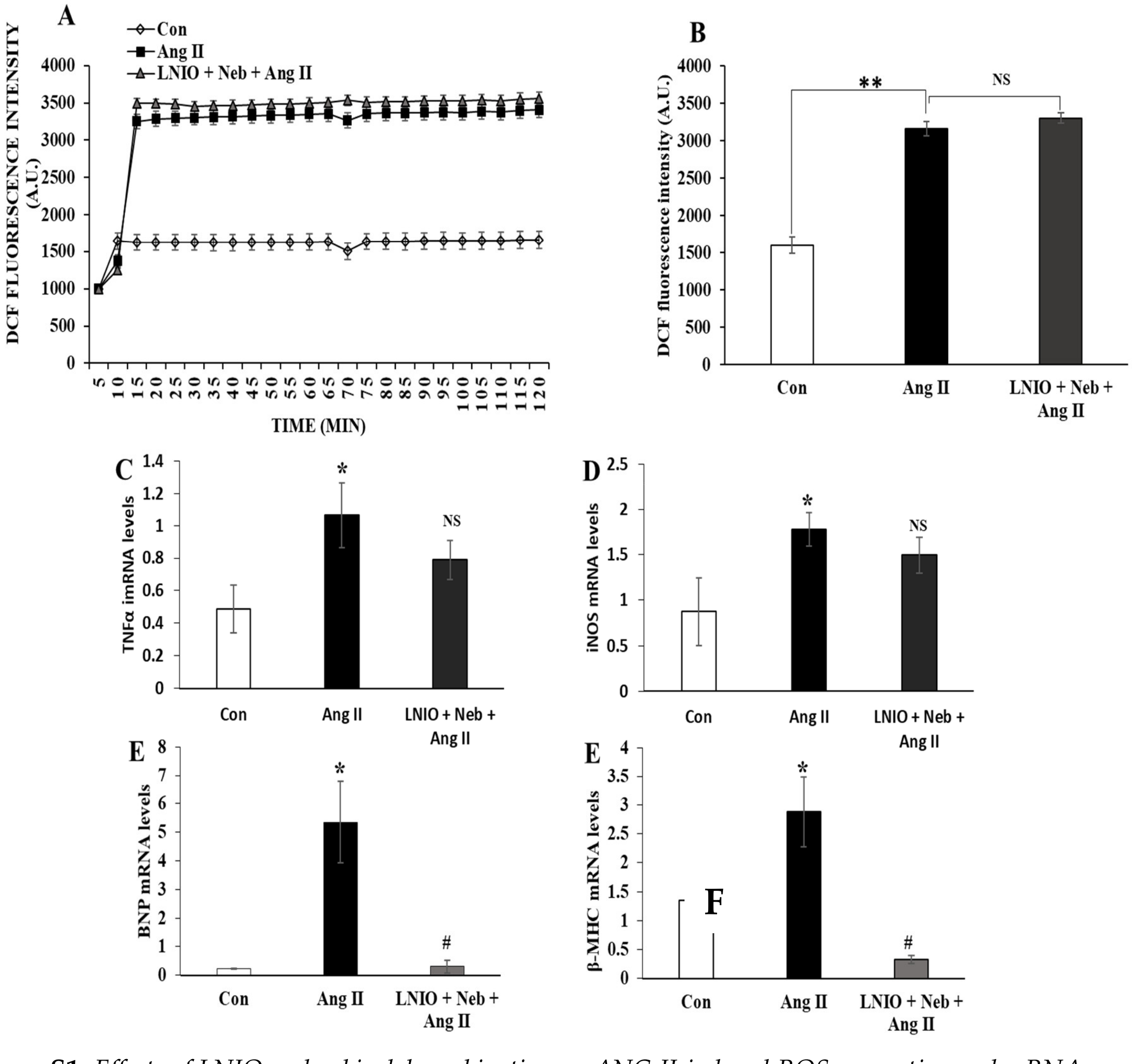

Figure S1. *Effects of LNIO and nebivolol combination on ANG II-induced ROS generation and mRNA expression of hypertrophy and inflammatory genes.* ANG II, induced increase in DCFDA fluorescence was not affected by pretreatment with *LNIO* plus Neb (A, B). ANG II-mediated mRNA expression levels of proinflammatory cytokines TNF-α (C) and iNOS (D) were not reduced by LNIO plus Neb pretreatment. ANG II-mediated mRNA expression of hypertrophy markers BNP (E) and β-MHC (F) were significantly reduced by LNIO plus Neb pretreatment. $**P < 0.02$, $*P < 0.05$ vs. untreated (Con), $\#P < 0.05$ vs. ANG II and NS, nonsignificant vs. ANG II. Values are means ± SEM N ≥ 6 for each treatment group
